# A290 ILLICIT SUBSTANCE USE AND ITS IMPACT ON ALCOHOL-ASSOCIATED HEPATITIS IN LATIN AMERICA

**DOI:** 10.1093/jcag/gwad061.290

**Published:** 2024-02-14

**Authors:** A H Islam, L A Diaz, F Idalsoaga, G Ayares, J Arnold, B Alcayaga, K Maldonado, J Arab

**Affiliations:** London Health Sciences Centre, London, ON, Canada; Pontificia Universidad Catolica de Chile, Santiago, Chile; Pontificia Universidad Catolica de Chile, Santiago, Chile; Pontificia Universidad Catolica de Chile, Santiago, Chile; Pontificia Universidad Catolica de Chile, Santiago, Chile; Pontificia Universidad Catolica de Chile, Santiago, Chile; Hospital Roosevelt de Guatemala, Guatemala, Guatemala, Guatemala; London Health Sciences Centre, London, ON, Canada

## Abstract

**Background:**

Concomitant substance use is frequent among patients with alcohol use disorder (AUD), but its impact on alcohol-associated hepatitis (AH) is unknown.

**Aims:**

To assess the prevalence and impact of substance use in patients hospitalized for AH in a multinational cohort in Latin America.

**Methods:**

Multicenter prospective cohort study including patients with AH between 2015-2022. We recorded sociodemographic and clinical information, including data on alcohol and drug use. We assessed the impact of substance consumption using competing-risk models.

**Results:**

We included 405 patients from 24 centers in 8 countries (Argentina, Bolivia, Brazil, Chile, Colombia, Ecuador, Mexico, and Peru). The mean age was 49.6±12.2 years, 345 (85.4%) were men, 210 (57.5%) had a previous diagnosis of cirrhosis, and the median MELD at diagnosis was 25 [20–31] points. Around 74% of patients fulfilled ACLF criteria (ACLF-1: 11.1%, ACLF-2: 11.6%, ACLF-3: 49.6%). A total of 82 (20.3%) reported active substance use, while 22 (5.4%) were former substance users. The most common drugs used at admission were marijuana (11.1%), cocaine (10.4%), methamphetamine (4.4%), and heroin (0.5%). Out of the total, 35.7% died, and only 2.5% underwent liver transplantation during follow-up. Active substance use was higher in younger patients (users 44.4±16.1 years vs. non-users 51.0±10.6 years; pampersand:003C0.001) and in men compared to women (22.0% vs 10.2%, p=0.036). In a competing-risk model adjusted by age, sex, history of cirrhosis, MELD, and ACLF grade, active substance use was independently associated with mortality (subdistribution Hazard Ratio [sHR] 1.53, 95%CI:1.01–2.32; p=0.043). Also, active cocaine (sHR 1.69, 95%CI:1.07–2.70; p=0.025) and marijuana use (sHR 1.83, 95%CI:1.11–3.04; p=0.018) were independently associated with mortality in adjusted competing-risk analyses.

**Conclusions:**

Active drug use is common in AH patients. Marijuana and cocaine were the most frequent substances and were independently associated with increased mortality. Substance use should be screened in patients with AUD, and integrated management with addiction specialists and psychiatrists could impact survival in AH.

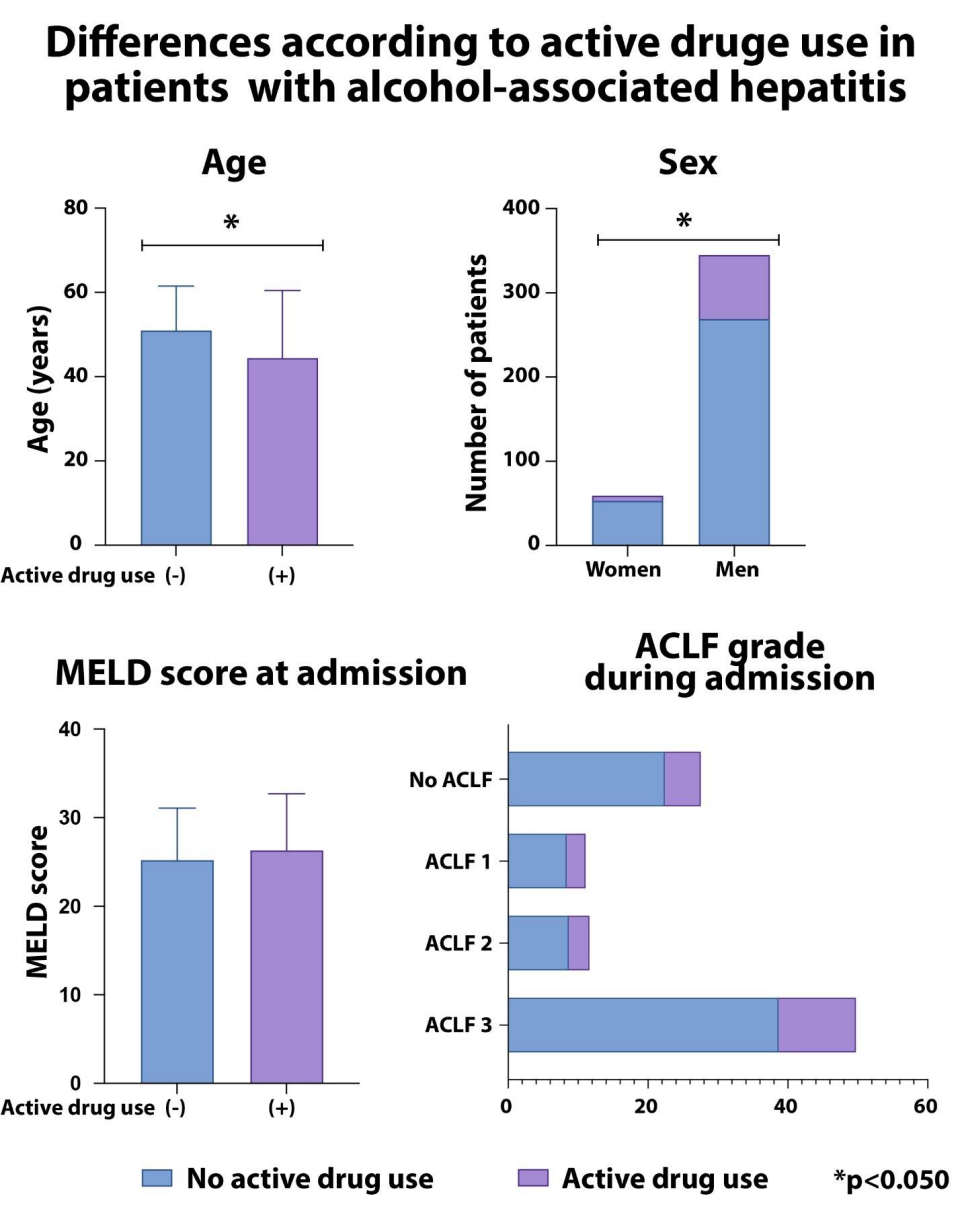

**Funding Agencies:**

None

